# Variation in depressive symptom trajectories in a large sample of couples

**DOI:** 10.1038/s41398-022-01950-w

**Published:** 2022-05-18

**Authors:** Zsófia Csajbók, Zuzana Štěrbová, Peter K. Jonason, Pavla Cermakova, Ádám Dóka, Jan Havlíček

**Affiliations:** 1 Faculty of Humanities, Charles University, Prague, Czech Republic; 2 Faculty of Science, Charles University, Prague, Czech Republic; 3 Faculty of Arts, Charles University, Prague, Czech Republic; 4 Department of General Psychology, University of Padua, Padua, Italy; 5 Institute of Psychology, Cardinal Stefan Wyszyński University, Warsaw, Poland; 6 Second Faculty of Medicine, Charles University, Prague, Czech Republic; 7 National Institute of Mental Health, Klecany, Czech Republic

**Keywords:** Depression, Human behaviour

## Abstract

The occurrence of depression is influenced by social relationships, however, most studies focus on individuals, not couples. We aimed to study how depressive symptoms of couples evolve over time and determine, which characteristics are associated with their distinct trajectories. A multi-centric cohort sample of 11,136 heterosexual couples (mean age = 60.76) from 16 European countries was followed for up to 12 years (SHARE study). Information on depressive symptoms measured by EURO-D scale was collected every 2 years. Dyadic growth mixture modeling extracted four distinct classes of couples: both non-depressed (76.91%); only women having consistently high depressive symptoms while men having consistently low depressive symptoms (8.08%); both having increasing depressive symptoms (7.83%); and both having decreasing depressive symptoms (7.18%). Couples with increasing depressive symptoms had the highest prevalence of relationship dissolution and bereavement. In comparison to the nondepressed class, individuals with any depressive symptoms were less psychologically and physically well. Our results suggest that distinct mechanisms are responsible for couples’ various longitudinal trajectories of depressive symptoms.

## Introduction

Depression is one of the most common mental disorders [1], adversely affecting people’s well-being, physical and mental health, and life expectancy [2, 3]. Estimates of its lifetime prevalence vary widely across countries (populational mean 13%) [1]. The prevalence of depression is more common in women [1], those with less education [4], and a lower socioeconomic status [5]. Depression is also related to social functioning, for example, lower relationship satisfaction [6], higher divorce rates [7], and adverse parenting performance [8, 9]. Importantly, the causality of these associations is unclear. For instance, is a lower relationship satisfaction the consequence or the source of depression? Further, social environments (e.g., the romantic partner) have diverse effects on depressive trajectories—they can play a protective role or serve as a mental health risk factor [10].

Positive assortative mating or homogamy (i.e., tendency to form intimate relationships with self-similar individuals) can be observed for socio-demographic (e.g., education and socioeconomic status), psychological (e.g., depression), and physical characteristics [11–13], even though the evidence is mixed [14]. Importantly, couples, where both are depressed, may be more likely to divorce [15]. While actor effects (i.e., the individual’s own depression) are better predictors of relationship difficulties than partner effects (i.e., the partner’s depression), the interaction of the actor and partner effects is important as well [6].

Homogamy between spouses can arise through various mechanisms [16], namely partner preferences for self-similarity, geographical and/or social proximity (i.e., social homogamy), convergence (i.e., a consequence of interactions and cohabitation), or secondary assortment (i.e., by-product of assortment in another characteristic, e.g., educational level and intelligence) [17]. Testing homogamy, however, has predominantly relied on cross-sectional data. This is problematic because data on couples’ similarity at one-time point indicates only a little about the causes of their homogamy, and nothing about their long-term similarity. From the perspective of interdependence theory [18], interacting partners affect each other’s experiences. One might expect partners’ mutual influence especially in relatively plastic traits (e.g., depressive symptoms) over time. In other words, the unit of analysis should be couples instead of individuals.

Depressive symptoms can change over time for many reasons (e.g., treatment, worsening of stressors, or their alleviation). Previously, four classes of aging individuals were found with different patterns of longitudinal depressive symptoms: consistently low, consistently high, increasing, and decreasing depressive symptoms [19, 20]. Thus, we may also expect that some couples have dynamically changing long-term dyadic patterns of depressive symptoms. Tracking spousal trajectories in depression is important to disentangle the dynamics and etiology of depression in a holistic perspective, considering the most important social factors (i.e., familial relationships). Importantly, we expect that not all couples will show the same trajectories, which can explain the difficulties in confirming the underlying mechanisms of assortative mating.

Differences in longitudinal depression trajectories can be explored by advanced analytical techniques. Here, in a six-wave prospective cohort study, the Survey on Health, Ageing and Retirement in Europe (SHARE), we tested the trajectories of assortative mating in depressive symptoms in long-term ageing couples, and its impact on their well-being, physical health, and relationship stability. Using the probabilistic growth mixture modeling approach, couples were divided into classes based on their longitudinal trajectories of depressive symptoms instead of using the classical correlation approach on the undivided total sample. This way, we could identify distinct classes of couples having differing longitudinal dyadic trajectories of depressive symptoms [21].

## Results

Altogether 11,136 couples made up the analytical sample. Homogamy was detected for age (*r* = 0.86, *p* < 0.001), education (*r* = 0.59, *p* < 0.001), and childhood socioeconomic position (*r* = 0.45, *p* < 0.001). After controlling for childhood socioeconomic position, we still found homogamy for education (*r* = 0.49; *p* < 0.001). In addition, homogamy was revealed for wellbeing at baseline (*r* = 0.53, *p* < 0.001), number of limitations in instrumental activities of daily living (*r* = 0.21, *p* < 0.001), number of chronic diseases (*r* = 0.27, *p* < 0.001), extraversion (*r* = 0.14, *p* < 0.001), agreeableness (*r* = 0.23, *p* < 0.001), conscientiousness (*r* = 0.24, *p* < 0.001), neuroticism (*r* = 0.15, *p* < 0.001), and openness (*r* = 0.28, *p* < 0.001).

**Fig. 1:**
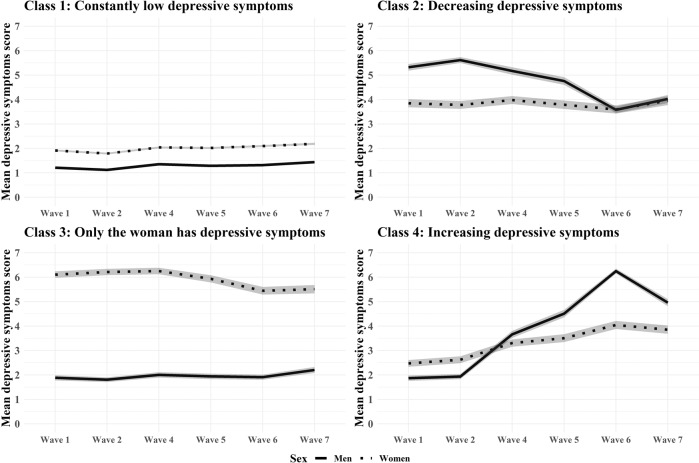
Dyadic latent trajectories of depressive symptoms across four classes of couples. Measurements taken every 2 years between Wave 1 and Wave 7, except in Wave 3; the scale of the depression scores ranged from 0 to 12; men’s and women’s mean depressive symptoms were presented in solid and dashed lines, respectively; 95% confidence intervals were shadowed in grey.

The dyadic growth mixture modeling suggested four classes of depressive symptoms (Fig. 1, Table 1). Class 1 was composed of consistently low depressive symptoms in both partners (76.91%). Class 2 was composed of couples with decreasing depressive symptoms (7.18%). Class 3 was composed of only women having consistently high depressive symptoms while men had consistently low depressive symptoms (8.08%). Class 4 was composed of couples with increasing depressive symptoms in both sexes (7.83%). Although in Class 2 both men and women had a decreasing level of depressive symptoms, men started at a higher level at baseline than their female partners and their rate of decline was greater than their partners’ (*p* < 0.001). In Class 4, men’s depressive symptoms were lower than women’s at baseline, but men’s symptoms increased more sharply (*p* < 0.001). Homogamy in the demographic variables only negligibly differed between the classes. Descriptive statistics of the classes are provided in Table 2 and further details of the analysis are in the supplementary information.

**Table 1:** Class proportions and mean intercept and slope results in the 4-class dyadic latent base growth model.

	*N* (%) of class members	Mean male latent intercept factor (95% CI)	Mean male latent slope factor (95% CI)	Mean female latent intercept factor (95% CI)	Mean female latent slope factor (95% CI)
**Class 1**	8565 (79.61%)	1.20 (1.14, 1.27)**	0.12 (0.02, 0.22)*	1.85 (1.75, 1.96)**	0.22 (0.05, 0.39)*
**Class 2**	799 (7.18%)	5.48 (5.11, 5.86)**	−1.44 (−1.88, −1.00)**	3.82 (3.50, 4.14)**	−0.20 (−0.49, 0.09)
**Class 3**	900 (8.08%)	1.82 (1.64, 2.01)**	0.07 (−0.13, 0.28)	5.91 (5.50, 6.32)**	−0.76 (−1.91, 0.40)
**Class 4**	872 (7.83%)	1.87 (1.38, 2.35)**	3.21 (2.70, 3.73)**	2.44 (2.15, 2.73)**	1.37 (1.07, 1.68)**

*CI* confidence interval.

*p < 0.05. **p < 0.001.

**Table 2:** Baseline characteristics of the participants across trajectories of depressive symptoms.

	Depressive symptoms				
	Class 1: Consistently low depressive symptoms	Class 2: Decreasing depressive symptoms	Class 3: Only the woman has depressive symptoms	Class 4: Increasing depressive symptoms	*η*^2^/*V*
Demographic variables					
Age, men, (*M*, *SD*)	61.86 (8.18)	63.82 (9.01)	62.79 (8.92)	64.81 (9.01)	0.012**
Age, women, (*M*, *SD*)	58.82 (8.40)	60.18 (9.21)	59.92 (9.14)	61.33 (9.31)	0.008**
Western Europe, *N* (%)	3496 (40.82)	267 (33.42)	309 (34.33)	307 (35.21)	0.056**
Southern Europe, *N* (%)	1823 (21.28)	207 (25.91)	306 (34.00)	272 (31.19)	0.099**
Scandinavia, *N* (%)	1086 (12.68)	39 (4.88)	51 (5.67)	63 (7.22)	0.091**
Central and Eastern Europe, *N* (%)	1825 (21.31)	238 (29.79)	200 (22.22)	185 (21.22)	0.053**
Israel, *N* (%)	335 (3.91)	48 (6.01)	34 (3.78)	45 (5.16)	0.031*
Education men, (*M*, SD)	3.01 (1.44)	2.49 (1.47)	2.43 (1.44)	2.53 (1.51)	0.023**
Education, women, (*M*, *SD*)	2.85 (1.43)	2.46 (1.51)	2.21 (1.43)	2.43 (1.47)	0.021**
Childhood SEP, men, (*M*, *SD*)	0.08 (1.59)	−0.45 (1.50)	−0.45 (1.42)	−0.39 (1.51)	0.018**
Childhood SEP, women, (*M*, *SD*)	0.15 (1.61)	−0.4 (1.57)	−0.41 (1.59)	−0.27 (1.72)	0.018**
*N* children, women, (*M*, *SD*)	2.36 (1.25)	2.59 (1.55)	2.48 (1.56)	2.44 (1.40)	0.003**
*N* grandchildren, women, (*M*, *SD*)	3.35 (3.24)	3.98 (3.55)	3.72 (3.63)	3.9 (3.66)	0.005**
Area of living, men, (*M*, *SD*)	3.51 (1.4)	3.54 (1.41)	3.57 (1.37)	3.45 (1.37)	<0.001
Area of living, women, (*M*, *SD*)	3.56 (1.4)	3.55 (1.41)	3.60 (1.41)	3.52 (1.42)	<0.001
Health variables					
Well-being, men, (*M, SD*)	38.96 (5.27)	31.82 (5.94)	36.14 (5.48)	35.12 (5.72)	0.131**
Well-being, women, (*M, SD*)	38.76 (5.35)	34.31 (6.19)	31.54 (6.35)	35.50 (6.07)	0.142**
Limitations of IADL, men, (*M, SD*)	0.08 (0.41)	0.47 (1.07)	0.14 (0.56)	0.25 (0.74)	0.04**
Limitations of IADL, women, (*M, SD*)	0.12 (0.47)	0.30 (0.73)	0.56 (1.08)	0.28 (0.74)	0.044**
*N* chronic diseases, men, (*M, SD*)	1.31 (1.26)	2.53 (1.80)	1.62 (1.47)	1.93 (1.49)	0.062**
*N* chronic diseases, women, (*M, SD*)	1.28 (1.28)	1.88 (1.59)	2.39 (1.66)	1.73 (1.53)	0.058**
Drugs for depression, men, *N* (%)	461 (5.38)	241 (30.16)	66 (7.33)	213 (24.43)	0.276**
Drugs for depression, women, *N* (%)	983 (11.48)	190 (23.78)	441 (49.00)	193 (22.13)	0.286**
Personality (Big Five) factors					
Extraversion, men, (*M, SD*)	3.46 (0.91)	3.26 (0.97)	3.41 (0.91)	3.24 (0.92)	0.006**
Extraversion, women, (*M, SD*)	3.55 (0.91)	3.47 (0.91)	3.32 (0.94)	3.50 (0.94)	0.005**
Agreeableness, men, (*M, SD*)	3.66 (0.81)	3.51 (0.87)	3.67 (0.83)	3.44 (0.87)	0.006**
Agreeableness, women, (*M, SD*)	3.75 (0.79)	3.64 (0.85)	3.62 (0.86)	3.66 (0.80)	0.003**
Conscientiousness, men, (*M, SD*)	4.10 (0.79)	3.96 (0.85)	4.08 (0.77)	3.85 (0.86)	0.008**
Conscientiousness, women, (*M, SD*)	4.18 (0.75)	4.07 (0.79)	3.99 (0.81)	4.04 (0.85)	0.007**
Neuroticism, men, (*M, SD*)	2.44 (0.94)	3.04 (0.97)	2.48 (0.94)	3.09 (0.98)	0.047**
Neuroticism, women, (*M, SD*)	2.73 (1.02)	3.01 (1.01)	3.42 (1.00)	2.90 (1.05)	0.034**
Openness, men, (*M, SD*)	3.24 (0.93)	3.21 (0.97)	3.17 (0.92)	3.07 (1.01)	0.002**
Openness, women, (*M, SD*)	3.36 (0.95)	3.29 (0.96)	3.24 (0.98)	3.23 (0.95)	0.002**
Bereavement					
Death of man, *N* (%)	404 (4.72)	113 (14.14)	97 (10.78)	138 (15.83)	0.154**
Death of woman, *N* (%)	187 (2.18)	28 (3.50)	50 (5.56)	56 (6.42)	0.084**
Break-up, *N* (%)	170 (1.98)	22 (2.75)	33 (3.67)	37 (4.24)	0.048**

Effect sizes were expressed in eta-squared when comparing continuous variables and in Cramer’s V when comparing binary variables.

*M* mean, *SD* standard deviation, *SEP* socioeconomic position, *IADL* instrumental activity of daily living.

*p < 0.05; **p < 0.001.

When compared to couples that were consistently low on depressive symptoms (Class 1), both men and women had lower baseline well-being within the couples with mutually decreasing depressive symptoms (Class 2) as well as within the couples, where both partners had increasing level of depressive symptoms (Class 4). Only women had lower baseline well-being within the couples, in which only women had consistently high depressive symptoms, but men had consistently low level of depressive symptoms (Class 3). Results were similar when tested for the total number of chronic diseases and limitations in instrumental activities of daily living. The prevalence of bereavement was about 3–4 times higher in Classes 2–4 than in the nondepressed reference Class 1 (except for the death of women in Class 2 having decreasing depressive symptoms). The Class 4 (*χ*^2^(1) = 14.37, *p* < 0.001) and Class 3 (*χ*^2^(1) = 4.11, *p* = 0.043) couples had higher chance of break-up than Class 1 couples. See further differentiating factors between the classes in Table 2 and the detailed results of the multinomial regression in Supplementary Table S8.

## Discussion

Depression affects various aspects of people’s lives. Here, we explored romantic couples in relation to their mutual trajectories of depressive symptoms and how those trajectories were associated with relationship stability, bereavement, and well-being. In a large sample of couples from Europe, we identified four classes of couples based on their dyadic longitudinal depression trajectories. Most couples (76.91%) were homogamous in their low levels of depressive symptoms; followed by a class, where only women had high depressive symptoms (8.08%, i.e., heterogamy); 7.83% of couples with increasing symptoms; and 7.18% of couples with decreasing symptoms. Further, we observed that couples were strongly homogamous in age, level of education, well-being, and childhood socioeconomic position, and were moderately homogamous in physical health and personality.

Notably, the couples with increasing depressive symptoms (Class 4) had the same direction of change (i.e., both increasing) but different rates of change and different endpoints of mean depressive symptoms. The couples with decreasing depressive symptoms (Class 2) also had the same direction of change (i.e., both decreasing), but they had different starting points converging to similar ending points. Using the traditional nomenclature [11], we may call the second group “convergent”, but the first group could be both labeled “divergent” and “convergent”. Even though the direction of change was the same in both partners in both classes, their levels of depression at the end of data collection were different. The traditional nomenclature of couples’ divergence only considered couples having similar starting levels and different ending levels in a characteristic over time. However, importantly, we added another dimension to categorizing couples’ joint longitudinal changes, the direction of change. We argue that true divergence would mean having different directions of change over time (i.e., one partner increasing in depressive symptoms, the other decreasing in symptoms). According to this rationale, we did not identify any class of couples truly divergent in depressive symptoms.

Why is couples’ assortment in depression important? Evidence suggests at least partial genetic background together with familial effects, and especially with unique environmental effects in depressive disorders [22, 23]. Further, the offspring of parents who were concordant in affective disorders were at higher risk for depression than children with only one affected parent [24]. Thus, spousal similarity can rise the prevalence of depression in the offspring because of both genetic and environmental factors and their interactions [25]. Knowing the different patterns couples show in their depression trajectories, it would be possible to better explore how different relationships affect their offspring’s mental health.

When both partners had increasing depressive symptoms (Class 4), they had the highest prevalence of bereavement and relationship dissolution during subsequent assessment in comparison to the other classes, perhaps unsurprisingly. Importantly, although men’s rate of increase in their depressive symptoms was higher than women’s, both had lower well-being, and worse physical health than the nondepressed Class 1. A reasonable explanation could be that their diminishing health puts too much weight on these couples jeopardizing their relationship stability (either by death or dissolution). However, one would need to have a more detailed picture of their day-to-day lives including their psychological coping and living circumstances to be able to better plan possible intervention programs for these couples.

Couples in the increasing (Class 4) and decreasing (Class 2) depression classes have a certain synchronization in the direction of their depressive symptoms. However, in both classes, men had higher depressive symptoms in almost all waves than women. Interestingly, in the class where women’s depressive symptoms were consistently high, men were not affected by their partner’s symptoms. This is in accordance with recent findings that women were more susceptible to the emotional contagion of sadness than men (while no sex difference was found in happiness) [26]. Apart from the lack of emotional contagion in male partners, other underlying factors may have been responsible, for example different manifestations or types of depressive symptoms in each class.

The most common class consisted of both nondepressed, homogamous partners. Remarkably, on the other hand, there was no class consisting of both partners with consistently high depressive symptoms. We speculate that people with mutually high depression probably do not initiate a relationship with each other, or these couples are not stable, and their relationship quickly dissolute. However, we cannot rule out the latter possibility from these data, as couples should be followed-up from the initiation of their relationships.

### Future directions

Future research should investigate whether initial assortment and preference for low level of depressive symptoms is responsible for homogamy in having no depressive symptoms. Alternatively, part of these couples started heterogeneously, but after an adjustment period they both converged to have low depressive symptoms. Presently, these possibilities cannot be tested because our sample only consisted of established couples, and we have no data available about their depressive symptoms before their relationship started or from the beginning of their relationship.

In addition, our observations were limited to the study being collected in waves at 2-year intervals. Hence, day-to-day mood changes could not be detected, but instead, our study extracted trait-like, long-term patterns. To better understand the mechanisms of these trajectories, one should investigate in more detail the day-to-day dyadic dynamics within the couples including a more detailed analysis concerning the specific time of bereavement and relationship dissolution. This would allow researchers to explore who affects depressive symptoms, which is especially interesting in couples with increasing and decreasing depression. Targeting couples with similar directions of change would unfold the underlying shared environmental factors affecting both partners—even if the effect is differing in degrees between the sexes.

## Limitations and conclusions

Evidence for homogamy in affective disorders is mixed [24]. There were methodological differences across previous studies, which may be responsible for that (e.g., depression criteria and measures used; hospital vs common population samples). This study is based on a cross-national, longitudinal, and representative sample. Such data are unparalleled in relationship research and couples’ data. Although we did find some differences between the classes apart from their depressive symptoms and well-being (e.g., region of origin), we did not predict them, and thus, they deserve more attention in the future. As already mentioned above, the two-year time lag may be considered too large to observe mood-related fluctuations, thus our findings apply only to trait-like longitudinal patterns of change.

Further, we know little about important aspects of their relationships and mental health, which imposes constraints on the conclusiveness of our results. The couples were already established when the project started, and their overall low rates of relationship dissolution limited our ability to observe the effects of depression on relationship stability. Although these couples came from 16 European countries with varying cultural and economic backgrounds, they still share significant similarities in being Western, Educated, Industrialized, Rich, and Democratic [27] societies. As they were on average about 61 years of age, we cannot generalize these findings to younger couples either.

In sum, we showed different patterns in couples’ longitudinal trajectories of depressive symptoms. By defining four different classes, we suggest a new approach when considering assortative mating longitudinally. We detected factors, which were specific for each class, such as well-being, physical health, relationship dissolution, and bereavement rates. Consequently, the different classes of couples might need different intervention approaches. Lastly, we demonstrated that the classification approach and longitudinal data are useful both from the perspective of clinical practice and social sciences as they can differentiate unique longitudinal patterns of couples’ plastic characteristics. The study has an interesting setup for the future. It suggests that we tend to treat all couples as the same, which obscures important differences. Identifying between-couple differences is a new frontier of research. Not all couples are the same and not all factors will differ from couple to couple and not all factors which differ will matter.

## Materials and methods

### Participants

Data for the analysis came from a prospective cohort study SHARE, which was previously described in detail [28]. Briefly, SHARE is a multi-centric, multidisciplinary longitudinal study that was initiated with the aim to assess health, social network, and economic conditions of community-dwelling individuals in Europe and Israel. The first wave of SHARE was conducted in 2004, followed by five subsequent waves in ~2-year intervals: wave 2 in 2006/2007, wave 3 in 2008/2009, wave 4 in 2011/2012, wave 5 in 2013, wave 6 in 2015 and wave 7 in 2017.

Participants were sampled based on probability selection methods. To be eligible to take part, individuals must be at least 50 years old, speak the official language of the country, and not have lived abroad or in an institution at baseline. Data are collected using computer-assisted personal interviewing in the participants’ homes. In case the participants have a spouse, the spouses were also invited to take part, irrespective of their age. Therefore, the SHARE study is an optimal data source to study trajectories of depressive symptoms in couples.

This study was carried out in accordance with the Declaration of Helsinki. SHARE has been repeatedly reviewed and approved by the Ethics Committee of the University of Mannheim. All participants provided written informed consent. Data were pseudo-anonymized and participants were informed about the storage and use of the data and their right to withdraw consent.

### Analytical sample

We restricted the analysis to individuals who participated in SHARE with their spouses, and each had at least three measurements of depressive symptoms, irrespective of from which wave the data came. Inclusion of at least three measurements enabled us to study trajectories of depressive symptoms. From 206,723 individuals included in the SHARE database, 139,556 people completed at least one interview. From them, we identified 125,532 individuals who took part in SHARE with a spouse, therefore, there were 62,766 couples. We excluded 546 same-sex couples (1092 individuals) and 36,746 couples (73,492 individuals) that did not have at least three measures of depressive symptoms, leaving the final analytical sample of 11,136 couples (22,272 individuals). This sample size is sufficient to perform growth mixture modeling on data of six waves [29]. Mean age of men was 62.31 (SD = 8.42, ranged between 34 and 91) and of women was 59.21 (SD = 8.63, ranging between 24 and 90). Flowchart is presented in Supplementary Fig. S1.

### Trajectories of depressive symptoms

To assess depressive symptoms, SHARE uses the EURO-D scale, a tool that was originally developed to compare symptoms of depression in older adults across Europe [30] and has been used in multiple studies [31–33]. The measurement with EURO-D was conducted in all waves except for wave 3. Participants were asked whether they have experienced 12 symptoms during the last month (depressed mood, pessimism, suicidality, guilt, sleep, interest, irritability, appetite, fatigue, concentration, enjoyment, and tearfulness). For each symptom, they received one point, creating a scale ranging from 0 to 12 points, with higher values suggesting more depressive symptomatology.

Couples’ dyadic trajectories of depressive symptoms were extracted using dyadic growth mixture modeling in Mplus version 8.7. This approach combines the latent classification methods and latent growth modeling, which can thus differentiate latent trajectories of depressive symptoms. The couples that followed similar dyadic longitudinal trajectories at a high probability were grouped into the same latent classes. The final classification model selected for publication was performed according to the recent guidelines [21]. See the Supplementary information for all details of the performed analyses.

Following interpretation and supporting criteria, the 4-class model was selected for publication (see the 1-, 2-, 3-, 4-, and 5-class solutions in Supplementary Fig. S2). The 4-class model showed the following patterns of dyads: Class 1. Consistently low depressive symptoms in both members of the dyad (76.91%), Class 2. Decreasing level of depressive symptoms in both men and women (7.18%), Class 3. Only the women have consistently high depressive symptoms, but the men have consistently low depressive symptoms (8.08%) and Class 4. Increasing depressive symptoms in both members of the dyad (7.83%), see Fig. 1. The Figure was created in R Studio version 1.4.1717. In a subsequent analysis, the (4-class) latent classes were regressed in a multinomial regression on the covariates using the 3-step method. Break-up during the follow-up period as categorical distal outcome was predicted by the latent class variable using the DCAT method [34]. Mplus syntaxes can be obtained from the first author upon request.

### Covariates

Five sets of covariates were used in five respective models to study, which factors were associated with the trajectories of depressive symptoms. The first set included the following demographic variables: age (years), sex (men/women), region (Western Europe/Southern Europe/Scandinavia/Central and Eastern Europe/Israel), education (categories based on International Standard Classification of Education; ISCED 1997) [35], childhood socioeconomic position (a composite measure of crowding ratio and number of books at home at age 10, as used in a previous study) [32], the number of children and grandchildren, and the type of participant’s residence area (a big city/the suburbs or outskirts of a big city/a large town/a small town/a rural area or village).

The second set comprised the following health variables: well-being (measured using the Control, Autonomy, Self-realization, and Pleasure; CASP-12 scale) [36], number of limitations in instrumental activities of daily living (measured with a modified 7-item tool) [37], chronic diseases (total number), and drugs for depression (defined by self-reported information on the use of drugs against depression or anxiety). A subsample of individuals who participated in the 7th wave had available information on the third set of covariates, the Big Five personality traits (i.e., Extraversion, Agreeableness, Conscientiousness, Neuroticism, and Openness), measured by the Ten-Item Personality Inventory [38]. The fourth model included the *death* of either men or women during the study [20]. The fifth model included a variable *break-up*, which was defined as a transition from being in a couple with the studied spouse to being single or being with another partner, irrespective of whether the original partners got back together.

Information about covariates was acquired at baseline, which is the wave data on EURO-D was first available, except for the variables for *drugs for depression* (defined as ever reporting the drugs during the duration of the study), *death* (defined as deceased at any time during the duration of the study), and *break-up* (defined as ever breaking up with the spouse during the duration of the study). Covariates of both sexes were used, except for the number of children and grandchildren. As these were too strongly correlated between men and women, we included this only for women to avoid collinearity. Means and proportions were compared between classes in SPSS version 26.

## Supplementary information

Supplemental Material
