# Supplementary material for: Variation in depressive symptom trajectories in a large sample of couples
Source: Transl Psychiatry. 2022 May 18;12:206. doi: 10.1038/s41398-022-01950-w (PMC9113986; doi:10.1038/s41398-022-01950-w)
Supplement: Supplementary file 1 — Supplemental Material [file 41398_2022_1950_MOESM1_ESM.docx]

**Variation in depressive symptom trajectories in a large sample of couples**

Zsófia Csajbók^*^, Zuzana Štěrbová, Peter K. Jonason, Pavla Cermakova, Ádám Dóka, & Jan Havlíček

*Corresponding author: Zsófia Csajbók, Ph.D., Faculty of Humanities, Charles University, Pátkova 5, Prague 8, 182 00, Czech Republic.

**Email:** [zsofia.csajbok@fhs.cuni.cz](mailto:zsofia.csajbok@fhs.cuni.cz)

**SUPPLEMENTARY INFORMATION**

**Materials and methods**

*Growth Mixture Modelling*

The dyadic latent trajectories of the couples were extracted using dyadic growth mixture modelling. The final latent trajectory model submitted to further analyses was selected based on the recommended guidelines ^1^ and following the procedures in Formánek, et al. ^2^, where latent trajectories were identified for depressive symptoms for individuals (not dyads). Full information maximum likelihood method (FIML) was used to estimate missing values as the default Mplus procedure. First, we compared three dyadic latent growth models’ (DLGM) fit against the data before the classification: a DLGM with latent intercept and slope factors (fixed to test linear growth) with correlated depressive symptoms in each wave between men and women. Subsequently, a dyadic latent growth curve model (DLGCM) with latent intercept, slope, and quadratic factor; and a dyadic latent basis growth model (DLBGM) with only intercept and slope factors but with the depressive symptoms freely loading onto the slope factor estimating the rate and shape of change completely freely (with the first time of measurement fixed at zero, and the last fixed at one). DLGCM yielded the best model fit, but the estimated mean of the quadratic latent factor was only 0.001 in men and 0.003 in women, neither of which were larger than expected by chance (see also Table S1). Eventually, the DLBGM model with freely estimated time scores was selected to enter the growth mixture model considering its good model fit and flexible abilities to capture any shape of curves in the distinct trajectories.

We extracted 2-, 3-, 4-, and 5-class models with the DLBGM model entered to the Growth Mixture Modelling. Each extracted class predicted the dyadic latent growth factors of a class of couples. As a default in Mplus, the growth factor variances were constrained to be equal across classes. The correlations between the latent growth factors and the depressive symptoms in each wave between men and women were estimated to be equal across classes. Unique correlations within each class were not possible to estimate because of estimation error. At least three sets of random starts were tested in each model and a fourth run using the LRT-starts option yielding reliable parametric bootstrapped likelihood ratio tests for each classification model. The final model considered for publication was chosen based on multiple factors. Each model was replicated using different random starts with success except for the 5-class model which could not yield non-negative latent variable covariance matrix. Each model run performed with different sets of random starts replicated the best log-likelihood. Similarly, other indicators of model quality were all replicated using distinct random starts and improved when extracting additional classes (i.e., Akaike Information Criterion, AIC; consistent Akaike Information Criterion, CAIC; Bayesian Information Criterion, BIC; and sample-size adjusted Bayesian Information Criterion, SSA BIC).

The entropy was acceptable in each model, though slightly decreased in the 4-class model in comparison to the 2- and 3-class models. The significant Vuong-Lo-Mendell-Rubin Likelihood Ratio Test (VLMR LRT), the significant Luo-Mendell-Rubin Adjusted Likelihood ratio test (LMR ALRT), and the significant parametric bootstrapped likelihood test all indicated better model solutions in the k-class versus in the k-1 class model is all the 1- to 5-class models (p < 0.001; Table S2). Therefore, the 4-class model for further analyses was chosen based on interpretability (Table S3; Figures S2a-e). The slope estimates in the final, 4‑class model is shown in the Supplementary Table S4, the correlations between the latent factors in the Table S5, and the correlations between male and female depressive symptoms at each time point in Table S6.

The 4-class model showed the following patterns of dyads: Class 1) Constantly low depressive symptoms in both members of the dyad (76.91%), Class 2) Decreasing level of depressive symptoms in both men and women (7.18%), Class 3) Only the women have constantly high depressive symptoms, but the men have constantly low depressive symptoms (8.08%) and Class 4) Increasing depressive symptoms in both members of the dyad (7.83%), see Figure 1. As the covariance coverage was low in the total sample, the 4-class model was replicated in a subsample with no missing data on depressive symptoms (*n* = 1,115). The sensitivity analysis on the fully covered sample yielded an interpretatively comparable solution, which indicates the robustness of the results extracted using FIML method on the total sample (Figure S2f). The 4-class model was replicated also on low data coverage (i.e., on a subsample of couples both having only 1-1 measurements, *n* = 39,212), which analysis resulted in comparable patterns (Figure S2g). As the time spent between two waves varied across couples (Table S7), we also replicated the analysis with individually specified time points using the “mixture random missing” Mplus analysis. The resulting 4-class model showed comparable patterns and class memberships to the freely estimated slope result (Figure S2h).

Multinomial regression predicted by the baseline covariates the class membership of the couples using the 3-step method. To keep sufficient power of the analysis and sufficient data coverage, the predictors were entered into the 3-step analysis in separate blocks of analysis (i.e., Model 1: demographic variables, Model 2: health variables, Model 3: Big Five factors, Model 4: death of either partner, and Model 5: break-up as distal outcome; Table S8). Entering the covariates into the model still allowed us to replicate the results of the 4-class model. Break-up during the follow-up period as categorical distal outcome was predicted by the latent class variable using the DCAT method ^3^.

| **Table S1.** Model fit of the 1-class latent growth model with fixed linear slopes; the 1-class latent growth cure model; and the 1-class latent base growth model with freed slopes on the total sample | | | | | | | | | | |
| --- | --- | --- | --- | --- | --- | --- | --- | --- | --- | --- |
| **Model** | **Estimated** | **Slopes** | **χ^2^(df)** | **RMSEA** | ***p*-close** | **CFI** | **TLI** | **SRMR** | **AIC** | **BIC** |
| 1-class latent growth | intercept, slope | fixed (linear) | 316.228(58) | 0.020 | [.018; .022] | 0.990 | 0.989 | 0.024 | 349063.481 | 349297.655 |
| 1-class latent growth curve | intercept, slope, quadratic | fixed quadratic | 180.700(45) | 0.016 | [.014; .019] | 0.995 | 0.992 | 0.015 | 348953.953 | 349283.261 |
| 1-class latent base growth | intercept, slope | freed | 245.576(50) | 0.019 | [.016; .021] | 0.992 | 0.990 | 0.021 | 349008.830 | 349301.547 |
| *Note.* RMSEA=root mean square of approximation; CFI=comparative fit index; TLI=Tucker-Lewis index; AIC=Akaike information criterion; BIC=Bayesian information criterion. | | | | | | | | | | |

| **Table S2.** Model parameters and results of the 2-, 3-, 4-, and 5-class latent base growth models | | | | | | | |
| --- | --- | --- | --- | --- | --- | --- | --- |
| ***N* of classes** | **Best Log-Likelihood** | **AIC** | **BIC** | **Entropy** | **VLM RLR** | **LMR ALRT** | **BLRT** |
| 2 | -173300.760 | 346691.519 | 347020.826 | 0.849 | *p* < 0.001 | *p* < 0.001 | *p* < 0.001 |
| 3 | -172642.097 | 345384.194 | 345750.090 | 0.852 | *p* < 0.001 | *p* < 0.001 | *p* < 0.001 |
| 4 | -172258.655 | 344627.310 | 345029.797 | 0.811 | *p* = 0.004 | *p* = 0.004 | *p* < 0.001 |
| 5^a^ | -171959.506 | 344039.013 | 344478.089 | 0.807 | *p* = 0.002 | *p* = 0.002 | *p* < 0.001 |
| *Note.* AIC=Akaike information criterion; BIC=Bayesian information criterion; VLM RLT=Vuong-Lo-Mendell-Rubin likelihood ratio test; LMR ALRT=Luo-Mendell-Rubin adjusted likelihood ratio test; BLRT=bootstrap likelihood ratio test.  ^a^ Negative variance of a latent variable. | | | | | | | |

| **Table S3.** Class proportions and mean intercept and slope results in the 4-class dyadic latent base growth model | | | | | | | |
| --- | --- | --- | --- | --- | --- | --- | --- |
|  | ***N* of class members** | **% of total *N*** | **Mean male latent intercept factor (95% CI)** | **Mean male latent slope factor (95% CI)** | **Mean female latent intercept factor (95% CI)** | **Mean female latent slope factor (95% CI)** |  |
| **Class 1** | 8565 | 76.91 | 1.20 (1.14, 1.27)** | 0.12 (0.02, 0.22)* | 1.85 (1.75, 1.96)** | 0.22 (0.05, 0.39)* |  |
| **Class 2** | 799 | 7.18 | 5.48 (5.11, 5.86)** | -1.44 (-1.88, -1.00)** | 3.82 (3.50, 4.14)** | -0.20 (-0.49, 0.09) |  |
| **Class 3** | 900 | 8.08 | 1.82 (1.64, 2.01)** | 0.07 (-0.13, 0.28) | 5.91 (5.50, 6.32)** | -0.76 (-1.91, 0.40) |  |
| **Class 4** | 872 | 7.83 | 1.87 (1.38, 2.35)** | 3.21 (2.70, 3.73)** | 2.44 (2.15, 2.73)** | 1.37 (1.07, 1.68)** |  |
| CI = confidence interval. * *p* < .05. ** *p* < .001 | | | | | | | |

| **Table S4.** Estimated loading of the depressive symptoms on the latent slope factor in the 4-class latent basis growth model | | |
| --- | --- | --- |
|  | **Estimated loadings of men (*SEM*)** | **Estimated loadings of women (*SEM*)** |
| **EURO-D Wave 1** | 0.000 (0.000) | 0.000 (0.000) |
| **EURO-D Wave 2** | 0.000 (0.000) | 0.070 (0.077) |
| **EURO-D Wave 4** | 0.485 (0.062)* | 0.511 (0.053)* |
| **EURO-D Wave 5** | 0.753 (0.068)* | 0.759 (0.050)* |
| **EURO-D Wave 6** | 1.308 (0.117)* | 1.157 (0.073)* |
| **EURO-D Wave 7** | 1.000 (0.000) | 1.000 (0.000) |
| *SEM* = standard error of the mean.  * *p* < .001 | | |

| **Table S5.** Correlation between the latent growth factors | | | | | |
| --- | --- | --- | --- | --- | --- |
|  |  | 1 | 2 | 3 | 4 |
| **1** | **male intercept latent factor** | − |  |  |  |
| **2** | **male slope latent factor** | 0.020 | − |  |  |
| **3** | **female intercept latent factor** | 0.420** | -0.115 | − |  |
| **4** | **female slope latent factor** | -0.136* | 0.625** | -0.314 | − |
| * *p* < .05. ** *p* < .001 | | | | | |

| **Table S6.** Correlation between male and female depressive symptoms | |
| --- | --- |
| Depressive symptoms at wave 1 | 0.201* |
| Depressive symptoms at wave 2 | 0.198* |
| Depressive symptoms at wave 4 | 0.205* |
| Depressive symptoms at wave 5 | 0.240* |
| Depressive symptoms at wave 6 | 0.229* |
| Depressive symptoms at wave 7 | 0.343* |
| * *p* < .001 | |

| **Table S7**. Descriptive statistics and data coverage of the depression scores and the time spent between waves | | | | | | |  |
| --- | --- | --- | --- | --- | --- | --- | --- |
|  |  | *N* | Min. | Max. | Mean | *SD* | |
| Men | EURO-D Wave 1 | 4611 | 0 | 10 | 1.60 | 1.76 | |
|  | EURO-D Wave 2 | 6563 | 0 | 11 | 1.57 | 1.77 | |
|  | EURO-D Wave 4 | 9494 | 0 | 12 | 1.85 | 1.88 | |
|  | EURO-D Wave 5 | 9297 | 0 | 12 | 1.81 | 1.94 | |
|  | EURO-D Wave 6 | 9367 | 0 | 12 | 1.88 | 1.95 | |
|  | EURO-D Wave 7 | 4150 | 0 | 11 | 1.91 | 2.00 | |
| Women | EURO-D Wave 1 | 4586 | 0 | 12 | 2.44 | 2.19 | |
|  | EURO-D Wave 2 | 6516 | 0 | 12 | 2.37 | 2.21 | |
|  | EURO-D Wave 4 | 9503 | 0 | 12 | 2.62 | 2.22 | |
|  | EURO-D Wave 5 | 9385 | 0 | 12 | 2.56 | 2.24 | |
|  | EURO-D Wave 6 | 9539 | 0 | 12 | 2.62 | 2.30 | |
|  | EURO-D Wave 7 | 4415 | 0 | 12 | 2.71 | 2.41 | |
| Men | Days between Waves 1 and 2 | 4303 | 335 | 1765 | 903.64 | 214.70 | |
|  | Days between Waves 2 and 4 | 5125 | 1247 | 1978 | 1562.19 | 113.86 | |
|  | Days between Waves 4 and 5 | 8653 | 337 | 1096 | 716.85 | 94.72 | |
|  | Days between Waves 5 and 6 | 8395 | 457 | 1003 | 732.08 | 87.06 | |
|  | Days between Waves 6 and 7 | 8444 | 486 | 974 | 748.66 | 77.23 | |
| Women | Days between Waves 1 and 2 | 4276 | 335 | 1734 | 908.88 | 217.00 | |
|  | Days between Waves 2 and 4 | 5076 | 1249 | 1978 | 1561.87 | 115.29 | |
|  | Days between Waves 4 and 5 | 8678 | 337 | 1096 | 717.33 | 95.01 | |
|  | Days between Waves 5 and 6 | 8421 | 457 | 1003 | 730.80 | 86.84 | |
|  | Days between Waves 6 and 7 | 8533 | 517 | 974 | 749.40 | 76.72 | |
| *Note*. Min. = minimum. Max. = maximum. *SD* = standard deviation. | | | | | | |  |

| **Table S8** Associations of participants’ characteristics with trajectories of depressive symptoms (odds ratios with 95% confidence intervals for tests of categorical latent variable multinomial logistic regressions using the 3-step procedure) | | | | |
| --- | --- | --- | --- | --- |
|  | **Depressive symptoms** | | | |
|  | **Constantly low depressive symptoms** | **Decreasing depressive symptoms** | **Only the woman has depressive symptoms** | **Increasing depressive symptoms** |
| **Model 1: demographic predictors** | Reference |  |  |  |
| Age, male |  | 1.039 (1.011; 1.069)** | 0.983 (0.955; 1.013) | 1.038 (1.006; 1.071)* |
| Age, female |  | 0.979 (0.953; 1.005) | 1.018 (0.988; 1.048) | 1.011 (0.980; 1.043) |
| Southern Europe |  | 1.068 (0.761; 1.500) | 1.578 (1.167; 2.134)* | 1.536 (1.119; 2.110)* |
| Scandinavia |  | 0.394 (0.209; 0.742)*** | 0.526 (0.278; 0.998)** | 0.618 (0.380; 1.005)* |
| Central and Eastern Europe |  | 1.511 (1.137; 2.008)* | 1.287 (0.931; 1.779) | 0.994 (0.720; 1.372) |
| Israel |  | 1.311 (0.787; 2.183) | 0.876 (0.379; 2.021) | 1.597 (0.933; 2.731) |
| Education, male |  | 0.813 (0.724; 0.913)*** | 0.891 (0.795; 0.999)* | 0.890 (0.799; 0.991)* |
| Education, female |  | 1.008 (0.896; 1.133) | 0.755 (0.659; 0.865)*** | 0.964 (0.862; 1.079) |
| Childhood SEP, male |  | 0.922 (0.829; 1.024) | 0.879 (0.786; 0.984)* | 0.899 (0.817; 0.990)* |
| Childhood SEP, female |  | 0.878 (0.783; 0.985)* | 0.944 (0.816; 1.091) | 1.012 (0.930; 1.102) |
| N of children, female |  | 1.107 (0.999; 1.226) | 0.972 (0.869; 1.087) | 0.963 (0.863; 1.075) |
| N of grandchildren, female |  | 1.028 (0.976; 1.082) | 0.982 (0.927; 1.040) | 0.993 (0.939; 1.051) |
| Area of living, male |  | 0.995 (0.867; 1.141) | 1.020 (0.877; 1.187) | 0.999 (0.888; 1.124) |
| Area of living, female |  | 0.933 (0.816; 1.066) | 0.968 (0.831; 1.129) | 0.982 (0.865; 1.115) |
| **Model 2: health predictors** | Reference |  |  |  |
| Well-being, male |  | 0.803 (0.782; 0.824)*** | 1.020 (0.993; 1.048) | 0.908 (0.889; 0.928)*** |
| Well-being, female |  | 0.951 (0.928; 0.974)*** | 0.793 (0.772; 0.814)*** | 0.933 (0.913; 0.954)*** |
| Limitations of IADL, male |  | 1.417 (1.177; 1.706)** | 1.034 (0.756; 1.413) | 1.287 (1.065; 1.556)* |
| Limitations of IADL, female |  | 1.095 (0.914; 1.312) | 1.431 (1.224; 1.671)*** | 1.234 (1.055; 1.444)* |
| N of chronic diseases, male |  | 1.506 (1.391; 1.630)*** | 1.037 (0.941; 1.143) | 1.255 (1.169; 1.347)*** |
| N of chronic diseases, female |  | 1.130 (1.037; 1.231)** | 1.401 (1.297; 1.514)*** | 1.125 (1.045; 1.211)** |
| Drugs for depression, male |  | 8.953 (6.543; 12.252)*** | 1.304 (0.783; 2.173) | 7.240 (5.482; 9.563)*** |
| Drugs for depression, female |  | 2.035 (1.514; 2.734)** | 7.939 (6.208; 10.154)*** | 1.984 (1.535; 2.566)*** |
| **Model 3: Big Five predictors** | Reference |  |  |  |
| Extraversion, male |  | 0.897 (0.761; 1.056) | 1.019 (0.884; 1.174) | 0.878 (0.750; 1.029) |
| Extraversion, female |  | 1.104 (0.945; 1.290) | 0.872 (0.757; 1.006)* | 1.146 (0.981; 1.339) |
| Agreeableness, male |  | 0.981 (0.825; 1.168) | 1.063 (0.900; 1.255) | 0.799 (0.673; 0.948)** |
| Agreeableness, female |  | 0.910 (0.756; 1.096) | 0.928 (0.782; 1.101) | 0.878 (0.746; 1.035) |
| Conscientiousness, male |  | 0.874 (0.735; 1.040) | 1.001 (0.851; 1.178) | 0.774 (0.652; 0.919)** |
| Conscientiousness, female |  | 0.903 (0.754; 1.082) | 0.716 (0.613; 0.835)*** | 0.828 (0.689; 0.996)* |
| Neuroticism, male |  | 2.008 (1.755; 2.297)*** | 0.892 (0.770; 1.032) | 2.326 (2.024; 2.672)*** |
| Neuroticism, female |  | 1.271 (1.110; 1.455)** | 2.403 (2.084; 2.771)*** | 1.120 (0.978; 1.283) |
| Openness, male |  | 1.076 (0.924; 1.252) | 0.943 (0.819; 1.086) | 0.889 (0.753; 1.049) |
| Openness, female |  | 0.991 (0.855; 1.149) | 0.992 (0.865; 1.136) | 0.820 (0.706; 0.952)** |
| **Model 4: bereavement** |  |  |  |  |
| Death of male | Reference | 4.246 (3.211; 5.615)*** | 3.020 (2.231; 4.089)*** | 4.708 (3.590; 6.725)*** |
| Death of female |  | 1.377 (0.797; 2.380) | 2.965 (1.978; 4.443)*** | 3.035 (2.017; 4.569)*** |
| **Model 5: break-up distal outcome** | Reference |  |  |  |
| Break-up |  | 1.598 (0.940; 2.716) | 2.223 (1.395; 3.544) | 3.015 (2.007; 4.529) |
| Due to listwise deletion of couples with missing observed predictors, the *N* of used observations was 7,229 in Model 1; 11,118 in Model 2; and 7,340 in Model 3. There was no missing predictor (neither outcome) in Models 4 nor 5, thus the *N* of used observations was 11,136. Results are odds ratio with 95% confidence intervals derived from multinomial logistic regression for the associations of participants’ characteristics with groups of couples with significant depressive symptoms, compared to couples who never had significant depressive symptoms. All participants’ characteristics were entered into the model simultaneously within each model. SEP = socioeconomic position. IADL = instrumental activities of daily living.  * *p* < .05, ** *p* < .01, *** *p* < .001 | | | | |

**
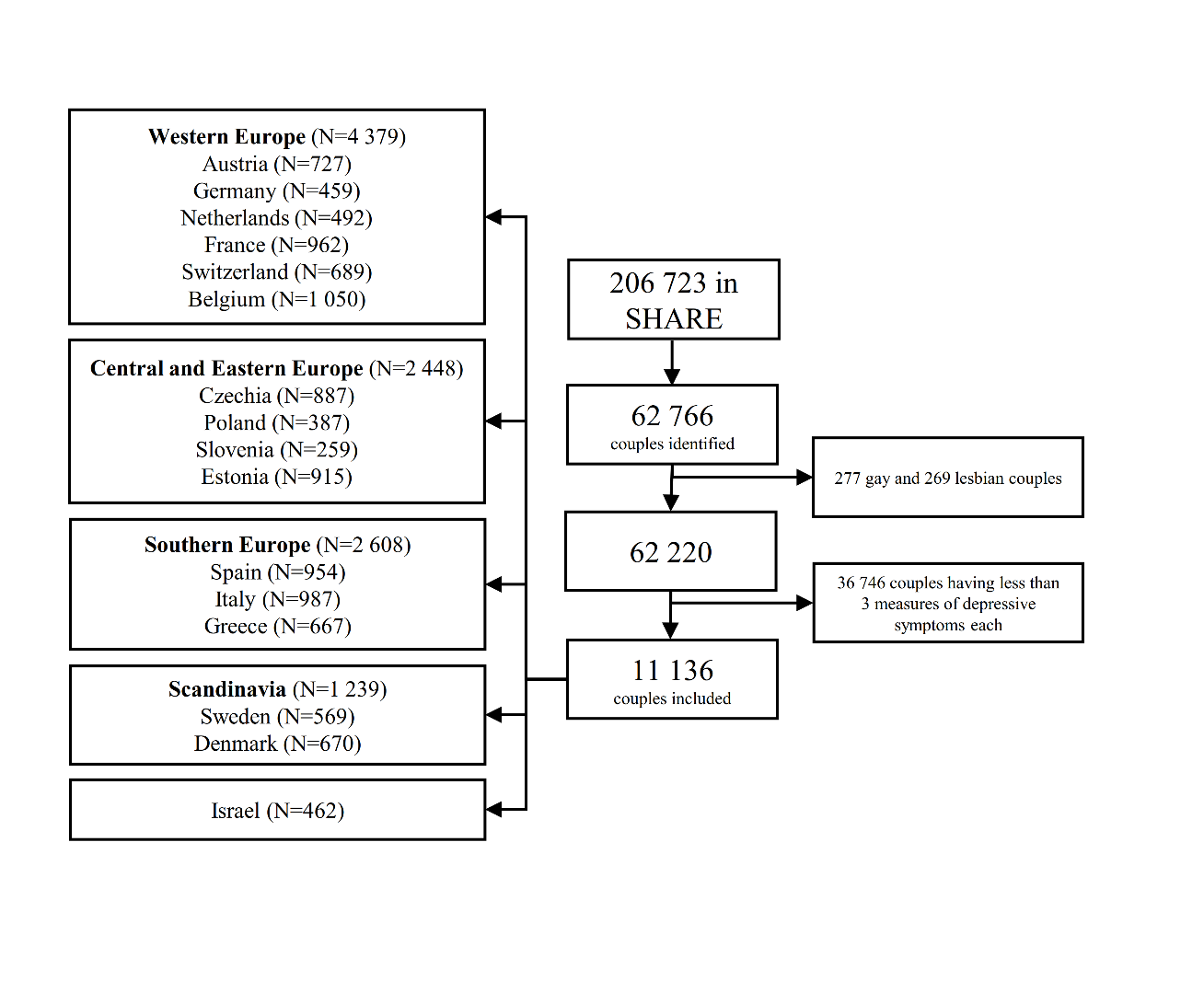
**

**Figure S1.** Selection of study participants

| **a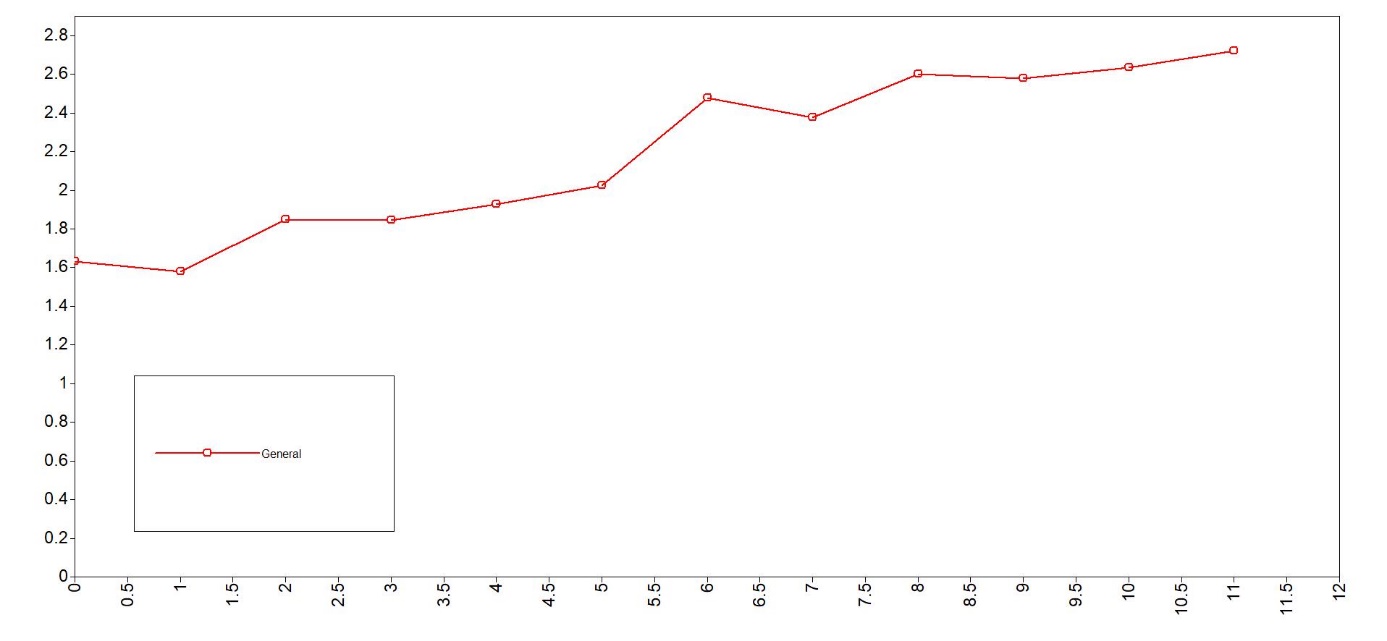** |
| --- |
| **b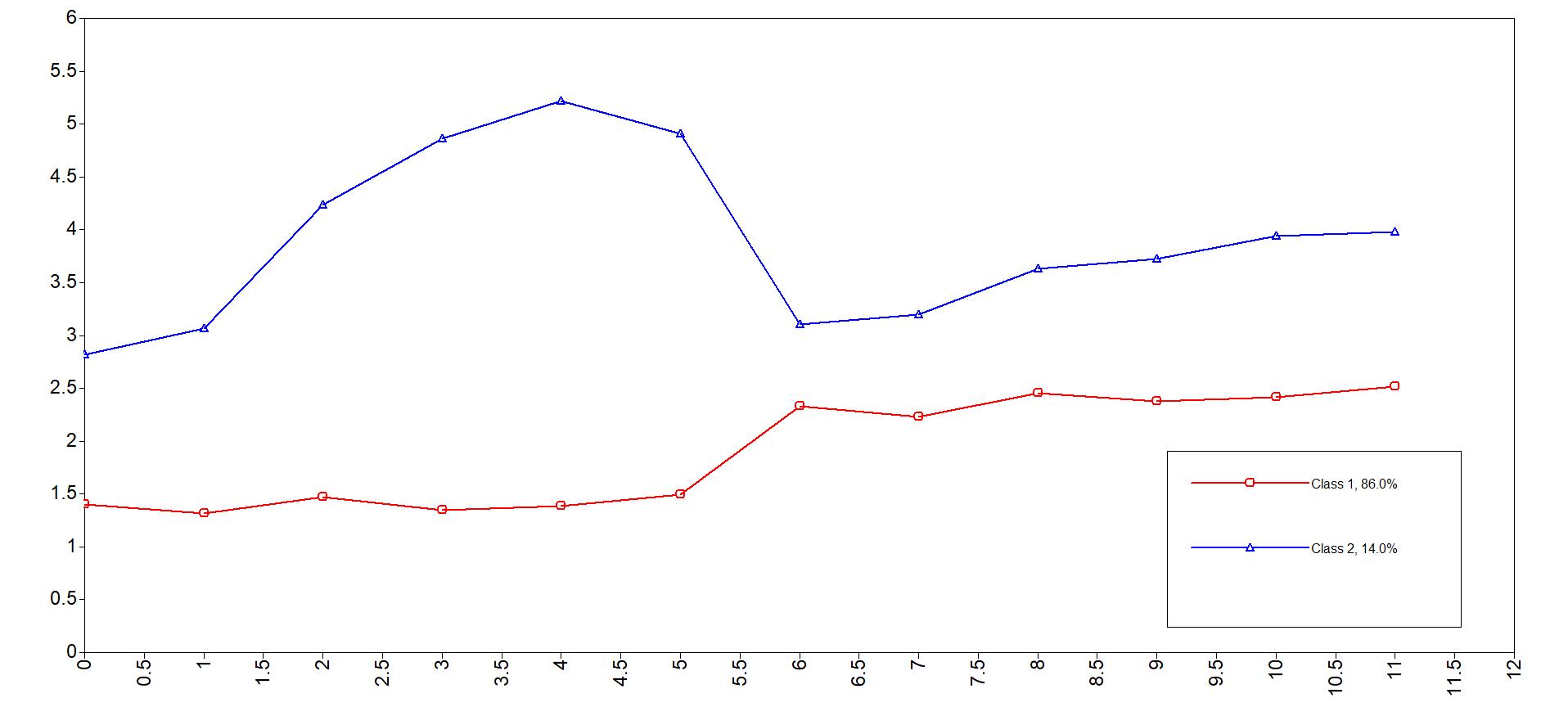** |
| **c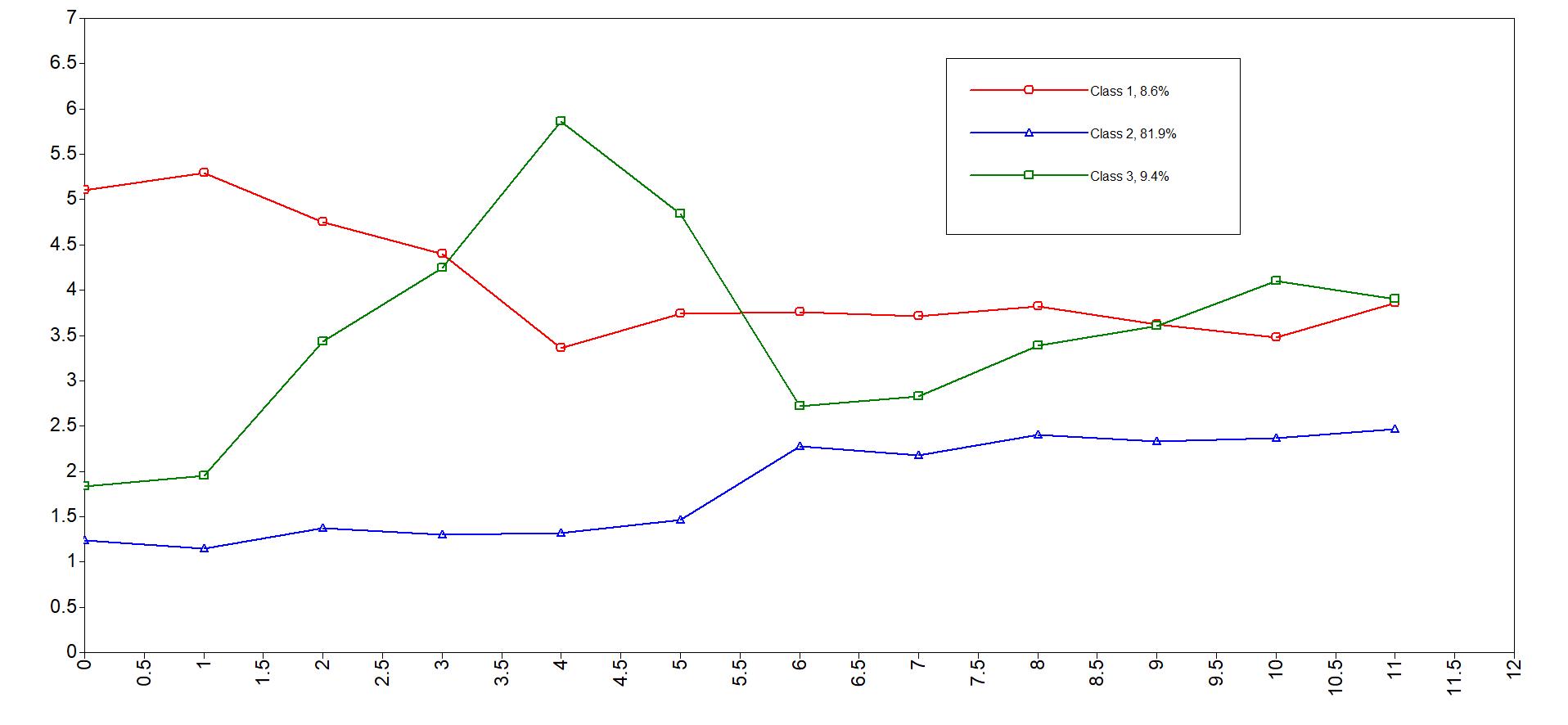** |
| **d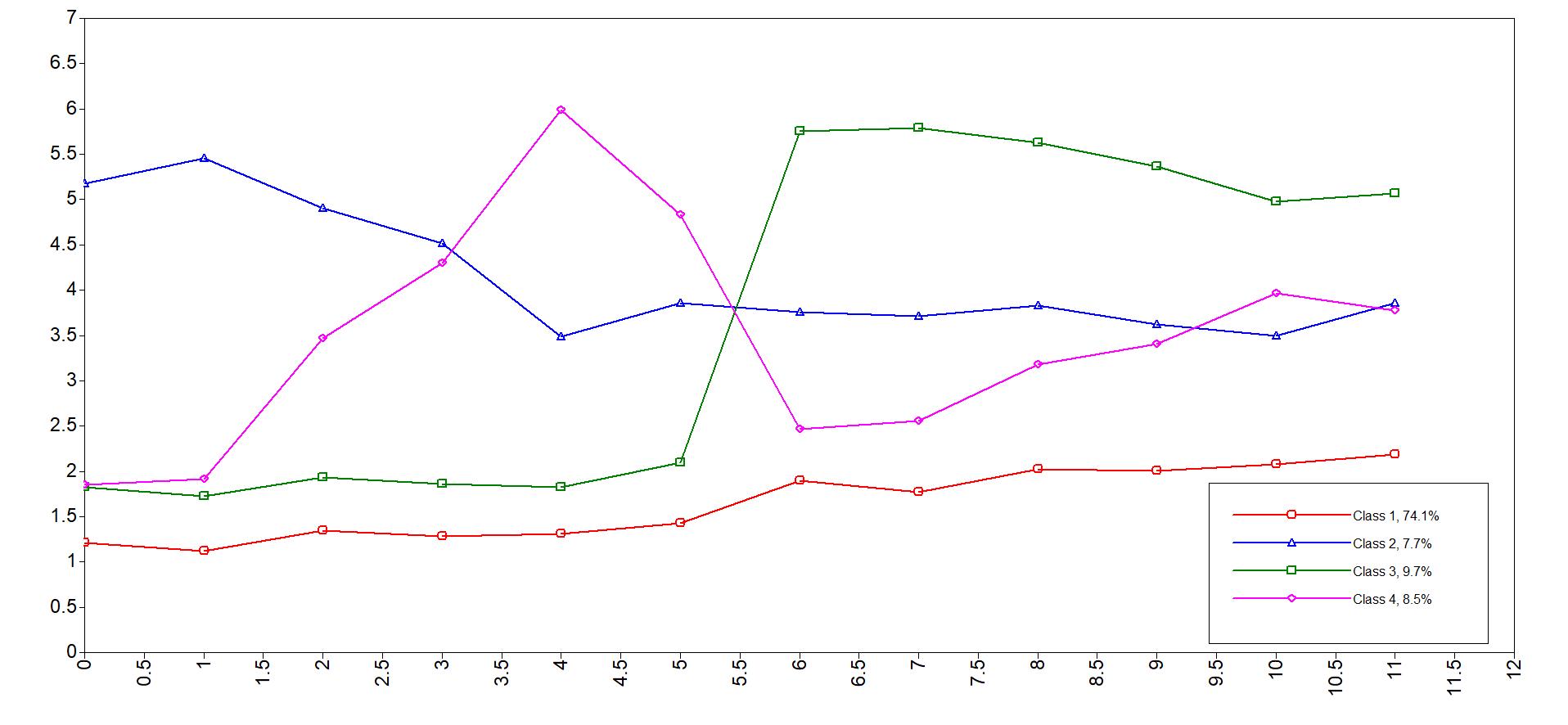** |
| **e** |
| **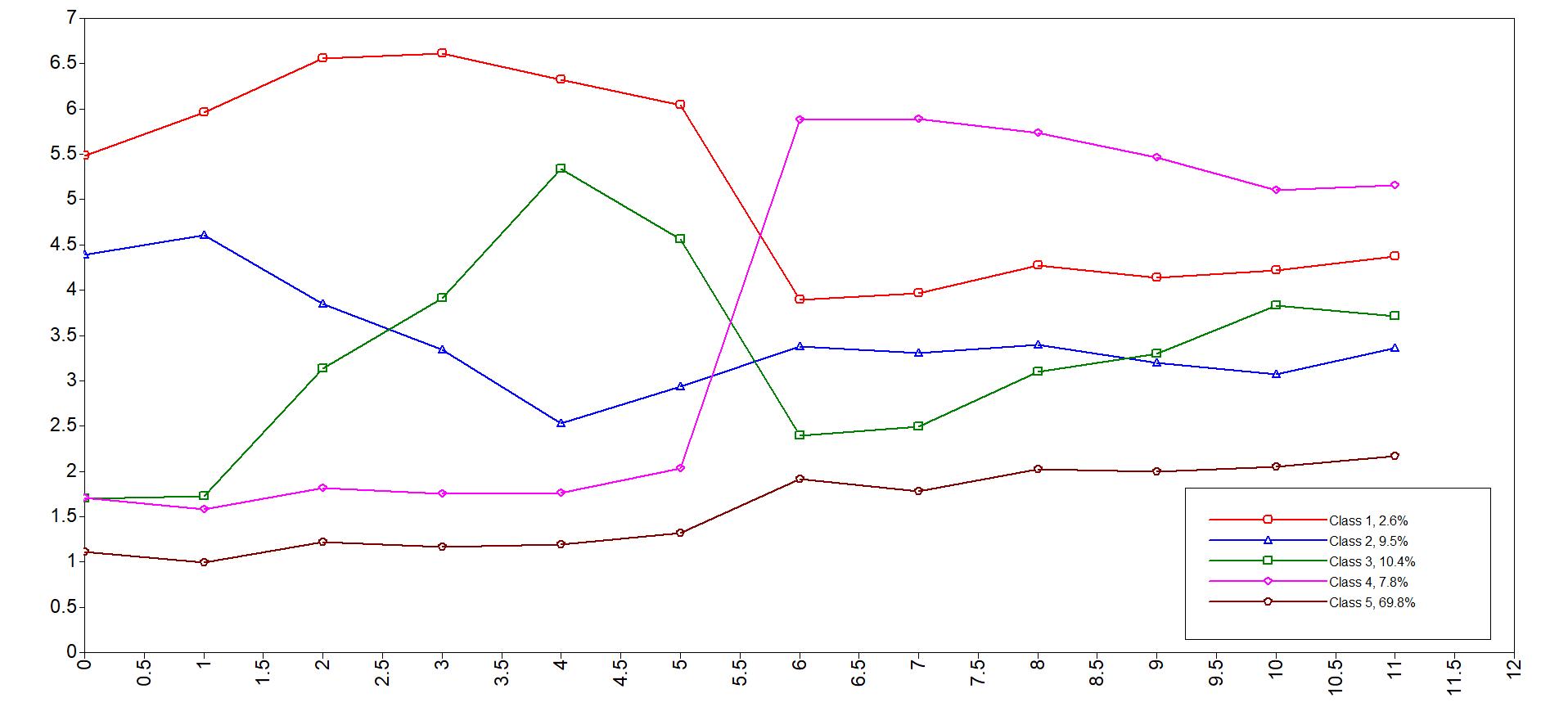f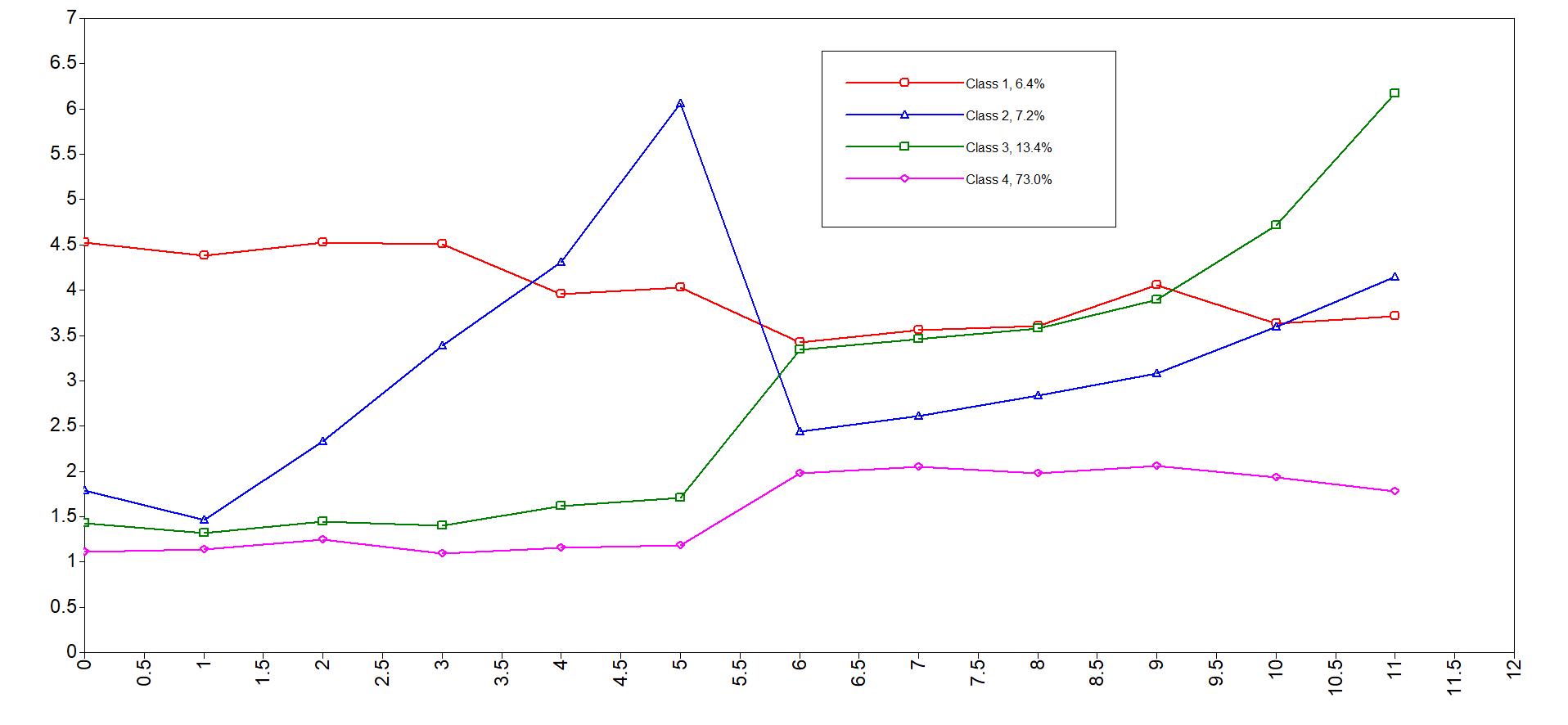** |
| **g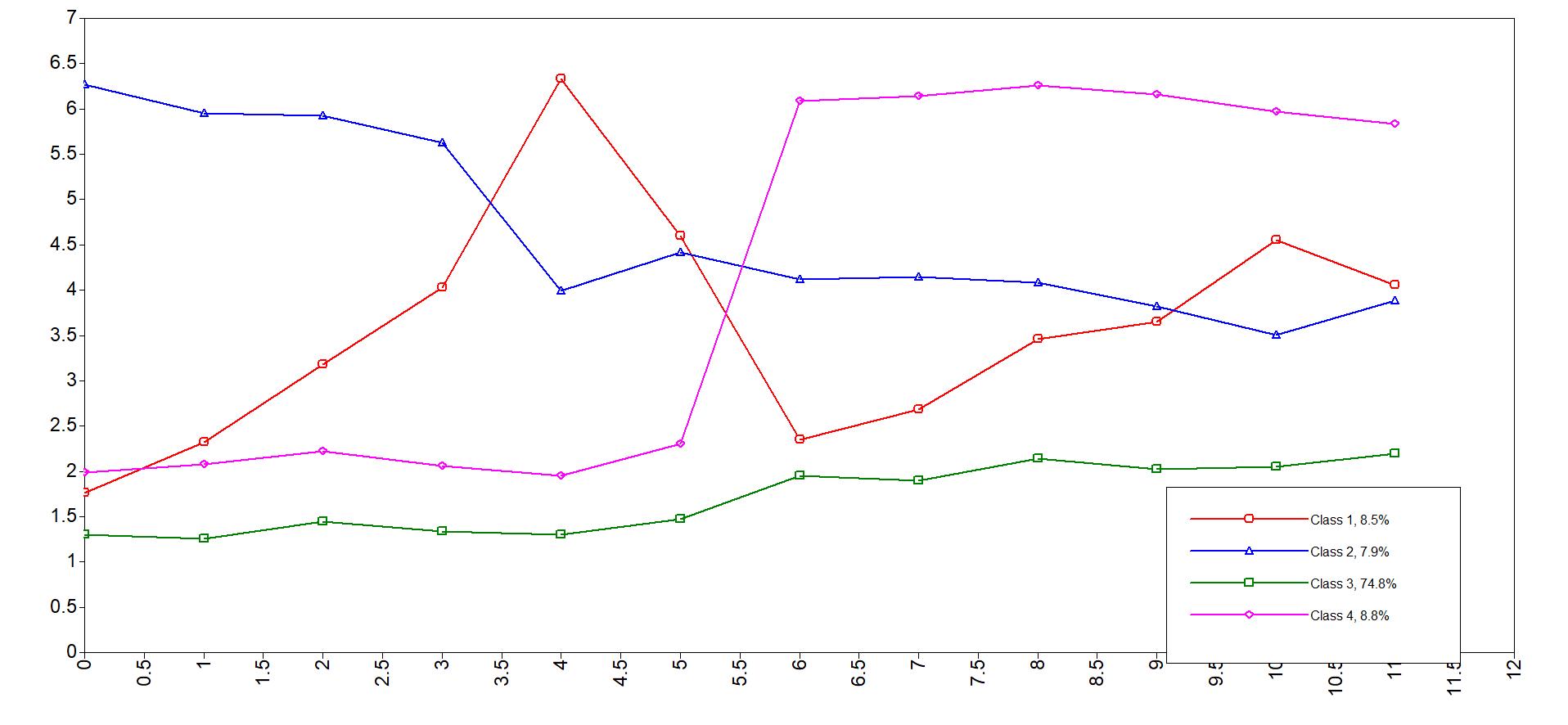** |
| **h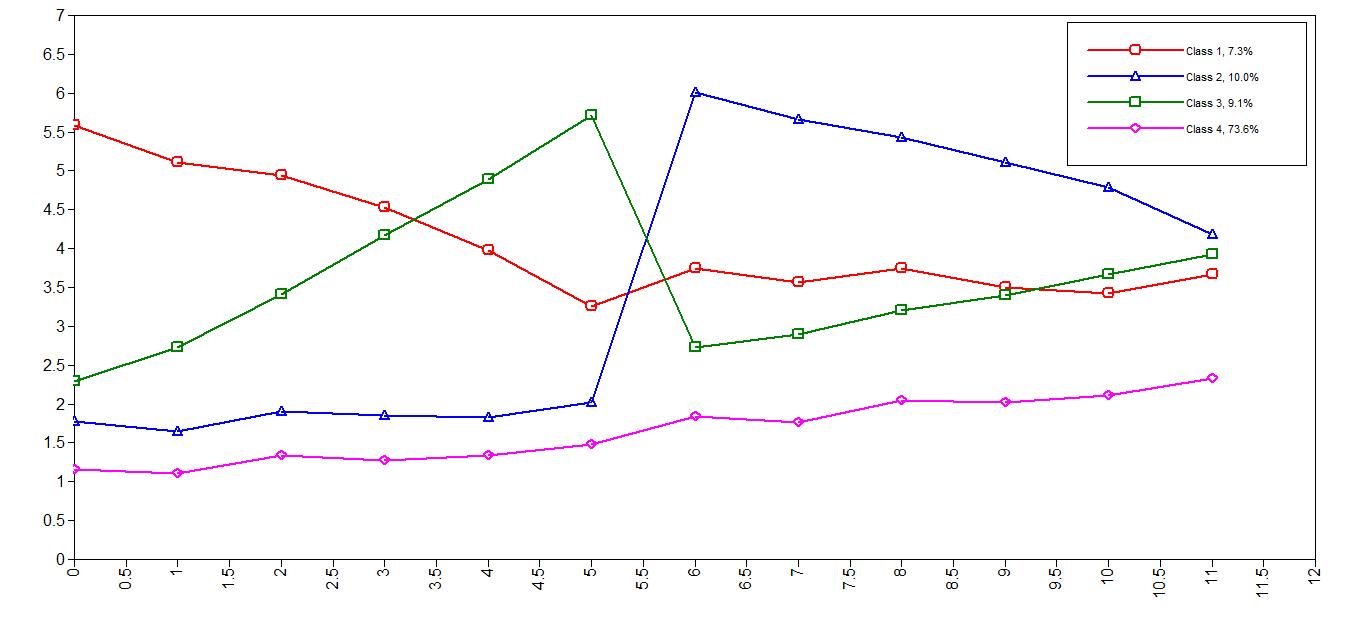** |
| **Figure S2.** Mean depressive symptoms of men and women (left: 0, 1, 2, 3, 4, and 5 points on the x axis; and right: 6, 7, 8, 9, 10, and 11 points on the x axis within each diagram, respectively) in each models extracting 1 to 5 classes. **a**: 1‑class model; **b**: 2‑class model; **c**: 3‑class model; **d**: 4‑class model; **e**: 5‑class model; **f**: 4-class model on full data coverage (i.e., on a subsample of couples both having all 6-6 measurements, *N* = 1,115); **g**: 4-class model on low data coverage (i.e., on a subsample of couples both having only 1-1 measurements, *N* = 39,212); **h**: 4-class model using individually specified time points (*N* = 10,740). |

**References**

1. van de Schoot R, Sijbrandij M, Winter SD, Depaoli S, Vermunt JK. The GRoLTS-checklist: Guidelines for reporting on latent trajectory studies. *Struct Equ Model* 2016; **24**: 451–467.

2. Formánek T, Csajbók Z, Wolfová K, Kučera M, Tom S, Aarsland D *et al.* Trajectories of depressive symptoms and associated patterns of cognitive decline. *Sci Rep* 2020; **10**: 1–11.

3. Lanza ST, Tan X, Bray BC. Latent class analysis with distal outcomes: A flexible model-based approach. *Struc Equ Model* 2013; **20**: 1.
